# Developmental Morphology, Physiology, and Molecular Basis of the Pentagram Fruit of *Averrhoa carambola*

**DOI:** 10.3390/plants13192696

**Published:** 2024-09-26

**Authors:** Wanli Tuo, Chunmei Wu, Xuexuan Wang, Zirui Yang, Lianhuan Xu, Siyuan Shen, Junwen Zhai, Shasha Wu

**Affiliations:** Key Laboratory of National Forestry and Grassland Administration for Orchid Conservation and Utilization at College of Landscape Architecture and Art, Fujian Agriculture and Forestry University, Fuzhou 350002, China; twl13395088391@163.com (W.T.); chumeiwu_7210@163.com (C.W.); candicewxx@163.com (X.W.); yzr12126@126.com (Z.Y.); lilianxuuu@163.com (L.X.); ssy13721075909@163.com (S.S.); zhaijw@163.com (J.Z.)

**Keywords:** *Averrhoa carambola*, pentagram fruit, morphological development, key regulatory genes, gene cloning

## Abstract

*Averrhoa carambola*, a key tropical and subtropical economic tree in the Oxalidaceae family, is distinguished by its unique pentagram-shaped fruit. This study investigates the developmental processes shaping the polarity of *A. carambola* fruit and their underlying hormonal and genetic mechanisms. By analyzing the Y1, Y2, and Y3 developmental stages—defined by the fruit diameters of 3–4 mm, 4–6 mm, and 6–12 mm, respectively—we observed that both cell number and cell size contribute to fruit development. Our findings suggest that the characteristic pentagram shape is established before flowering and is maintained throughout development. A hormonal analysis revealed that indole-3-acetic acid (IAA) and abscisic acid (ABA) show differential distribution between the convex and concave regions of the fruit across the developmental stages, with IAA playing a crucial role in polar auxin transport and shaping fruit morphology. A transcriptomic analysis identified several key genes, including *AcaGH3.8*, *AcaIAA20*, *AcaYAB2*, *AcaXTH6*, *AcaYAB3*, and *AcaEXP13*, which potentially regulate fruit polarity and growth. This study advances our comprehension of the molecular mechanisms governing fruit shape, offering insights for improving fruit quality through targeted breeding strategies.

## 1. Introduction

*Averrhoa carambola* is an important tree species that belongs to the genus *Averrhoa* of Oxalidaceae and holds significant ornamental and edible values. Its unique fruit exhibits a pentagram shape. It is commonly grown in gardens, along roadsides, within open forests, and in courtyards for its aesthetic appeal. It can also be grown in large pots on balconies or rooftops. In tropical and subtropical areas, it is often utilized as a fruit-bearing tree. Compared with other fruit trees, the *A. carambola* fruit has a peculiar shape and golden color, making it an important source to study the development of fruit shape. Studies on *A. carambola* mainly focus on germplasm resources and cultivation techniques [1,2], the genetic diversity of its fruit quality traits [3], and transcriptome analyses [4]. There are few studies on the formation mechanisms of the pentacle fruit shape of *A. carambola*.

Cell growth and development patterns underlie fruit shape variations [4,5]. Fruit morphology is shaped by internal pulp cell growth. Early cell division and later expansion primarily determine fruit size and shape [6]. In *Cucumis sativus*, rapid cell division occurs shortly after fertilization, followed by a slow growth until the cell size increases [7,8]. Some studies have shown that differences in fruit size and shape are mainly determined by the number of cells, such as *Cucumis melo* [9,10], *Cerasus avium* [11], *Prunus persica* [12], and *Pyrus* spp. [13]. In addition, the number and size of fruit cells are related to the final fruit size and shape, which is also the case in *Malus pumila* [14], cucumbers [15], *Vitis vinifera* [16], and *Ananas comosus* [17]. “Cell polarity” refers to the directional development of cells, tissues, or individuals, impacting their morphological and physiological characteristics [18]. Studies of *Arabidopsis thaliana* leaves unveiled distinctive features in the adaxial and abaxial mesophyll cells [19]. The *SUN* gene is a calmodulin-binding protein that can increase glucosinolate content, enhance defense ability, and regulate growth and development in *A. thaliana* [20,21]. The expression of the *SUN* gene in cotyledons, leaflets, flower organs, and ovaries was positively correlated with an elongated phenotype but negatively correlated with grain weight. The *SlSUN* gene was highly expressed in long-fruited tomatoes, but not in round-fruited tomatoes. The introduction of the *SlSUN* gene into a round-fruited tomato yields elongated and narrow fruits, and the knockout of the *SlSUN* gene produces round fruits [22]. Hormones such as auxin, gibberellin, cytokinin, abscisic acid, and ethylene play essential roles in the development of fruit shape [23,24]. Exogenous auxin (2,4-D) application affects fruit morphology by increasing the cell number and size in *Solanum lycopersicum* [25]. Auxin signaling genes, including *ARF*, *AUX*, and *IAA*, influence fruit development and morphology [26,27]. Transgenic *S. lycopersicum pAtPIN1* is crucial for the polar morphogenesis of tomato leaf primordia [28]. Auxin polar transport influences tobacco skin and fur cell elongation [29], suggesting its role in plant morphology. However, the role of auxin in *A. carambola* fruit shape variations remains underexplored.

*AUX*/*IAA* genes, common auxin-induced transcriptional suppressors, play central roles in auxin responses across plant organs [30]. The AUX/IAA transcription factor affects auxin polarity transport, leading to axial polarity defects and a narrow leaf width in the cucumber mf mutant [31]. *GH3* is an auxin response gene that encodes auxin amide synthetase, thereby promoting the amination of IAA and inhibiting the activity of auxin [32]. YABBY transcription factors regulate plant distal cell development, impacting lateral organ morphogenesis [33]. GH3 plays a key role in signaling pathways, organ development, and plant type [34,35]. Among them, *OsGH3.1*, *OsGH3.2*, *OsGH3.8*, and *OsGH3.13* are associated with the interaction between the IAA, JA, and SA signaling pathways under biotic and abiotic stresses [36,37,38]. The *YABBY* gene family is expressed in a polar manner [39]. At present, the *YABBY* gene has been reported in plant fruits, and the *YABBY* gene has been reported in *Vaccinium corymbosum* [40], *Citrus grandis* [41], *Vitis pseudoreticulata* [42], and *C. sinensis* [43]. But its role in fruit polarity regulation is unclear, especially in *A. carambola*.

Fleshy fruit development depends on morphological changes in flesh cells [40]. *XTH* genes regulate cell elongation and cell wall modification, affecting plant morphology [44]. Glycosyltransferases/hydrolases in xyloglucan, known as XTHs, play a crucial role in organ elongation by modifying xyloglucan chains [45]. *XTH* genes impact fruit softening [46,47], root development [48], and environmental stress responses [49]. The specific functions of *XTH* genes in *A. carambola* require further investigation.

Fruit shape is not only an economic feature of horticultural crops but also a key indicator of the ornamental value of plants [50]. Due to its distinctive geometric configuration, the pentagram shape offers a unique perspective on the spatial organization of cells and tissues during development. This configuration not only enhances our understanding of plant developmental processes but also serves as a representative model to investigate morphogenetic principles. Its symmetry and structural simplicity make it ideal for controlled experiments, offering insights into the genetic and molecular mechanisms of morphogenesis. Therefore, it is of great significance to explore the mechanism of fruit shape formation in plants. *A. carambola* fruit boasts a distinct pentagram shape, which is an ideal feature for studying fruit shape development. However, little is known about the mechanisms of the pentacle fruit shape of *A. carambola*. In this study, we explored the molecular mechanisms underlying the formation of the pentagram-shaped fruit in *A. carambola*. The morphological changes, hormone contents, and transcriptome data of *A. carambola* were ascertained. The key genes related to the growth and development of *A. carambola* pentagram fruit were screened, and their functions were preliminarily verified.

## 2. Results

### 2.1. Microscopic Observation of Six Developmental Stages of Bud and Fruit of A. carambola Blossom

**Figure 1:**
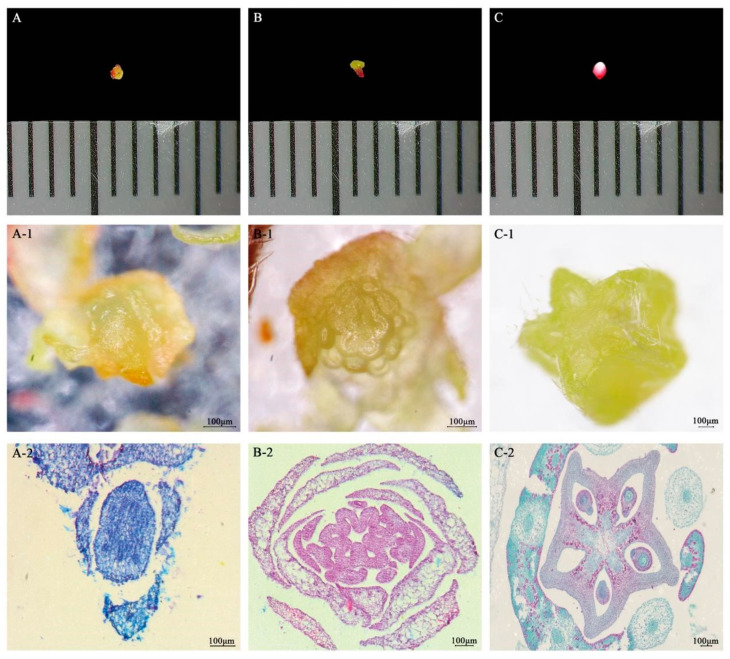
Observation on the pentacle ovary structure at three development stages of *A. carambola* flower bud: (**A**–**C**): microscopic view of the bud of *A. carambola* blossom; (**A-1**,**B-1**,**C-1**): visual micro-anatomical observation of the bud of *A. carambola* blossom; and (**A-2**,**B-2**,**C-2**): microstructure of paraffin section of *A. carambola* blossom.

**Figure 2:**
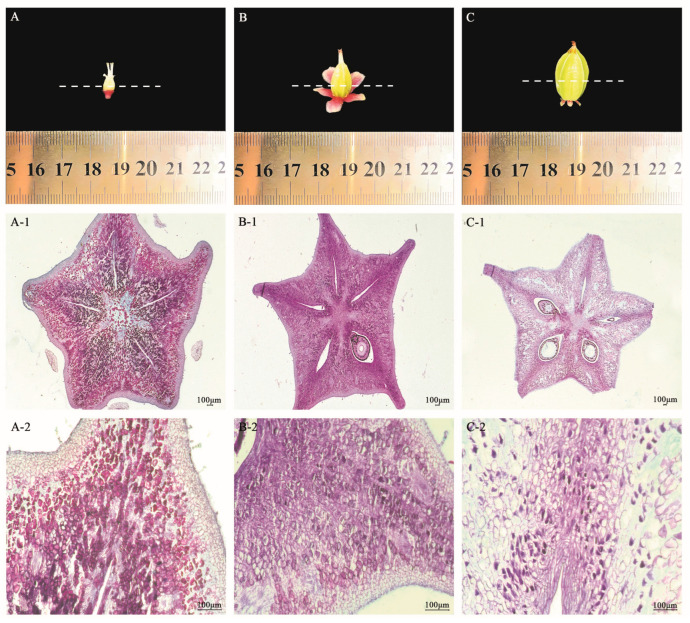

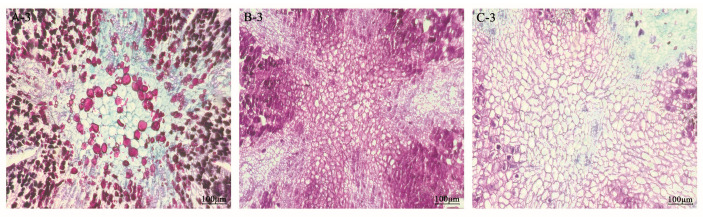
Observation of cell morphology in three developmental stages of *A. carambola* fruit: (**A**–**C**): microscopical view of the fruit of *A. carambola*; (**A-1**,**B-1**,**C-1**): microstructure of paraffin section of *A. carambola* fruit; (**A-2**,**B-2**,**C-2**): microstructure diagram of the convex of *A. carambola* fruit; and (**A-3**,**B-3**,**C-3**): microstructure diagram of the concave of *A. carambola* fruit.

The process of *A. carambola* bud differentiation was observed through a combination of microscope and paraffin sectioning. In the y1 stage of the bud, it was observed that the bud presented a light green color with no obvious pedicels and the ovary diameter was less than 1 mm. At this time, the petal primordia and sepals were formed (Figure 1A,A-1). From the details of the section, it was observed that the peripheral sepals had formed, and the appearance of the central part was mainly caused by local cell division in the clusters, resulting in the initial petal primordia and pistillate primordia. The overall area of the cells was small and densely arranged, and the ovary was nearly round (Figure 1A-2).

With the growth and development of the flower bud, entering the y2 stage, the diameter of the flower bud increased. The flower bud showed white–pink and red peels, while the ovary diameter was still less than 1 mm. The sepals, petals, stamen, and pistil primordium developed, and the embryo was shaped into a pentagonal star (Figure 1B,B-1). Through the sectioning, it is clearly seen that the sepals and petals were arranged in a wheel around the ovary, the stamen primordia and pistil primordia continued to develop, showing a clear outline of a pentagram, and the cells were densely arranged. Different from the first stage, the ovary in this stage showed the embryonic form of a pentagram (Figure 1B-2).

When the ovary developed to about 1 mm, which was in the y3 stage, the flower bud was red as a whole. The pedicel became green, and the volume was larger than the previous two stages. Here, the sepals, petals, stamens, and pistils were fully formed, and the ovary was enlarged, showing a three-dimensional pentagonal star shape (Figure 1C,C-1). The paraffin sectioning showed that the ovary was completely formed, and the corners of the pentagram were clearly visible. The embryo sac became more and more obvious with the development of the fruit and the ovary volume increased significantly. The cells were arranged neatly into squares. At the same time, the cells in the center were obviously larger than the peripheral cells, which was a characteristic of the cells in the division stage. Furthermore, vascular bundles were present around the axial placenta. It was evident that the ovary contained five ventricles, each of which housed one or two embryo sacs arranged around the axial placenta. The transverse section of the ovary exhibited a complete pentagonal shape. (Figure 1C-2).

**Figure 3:**
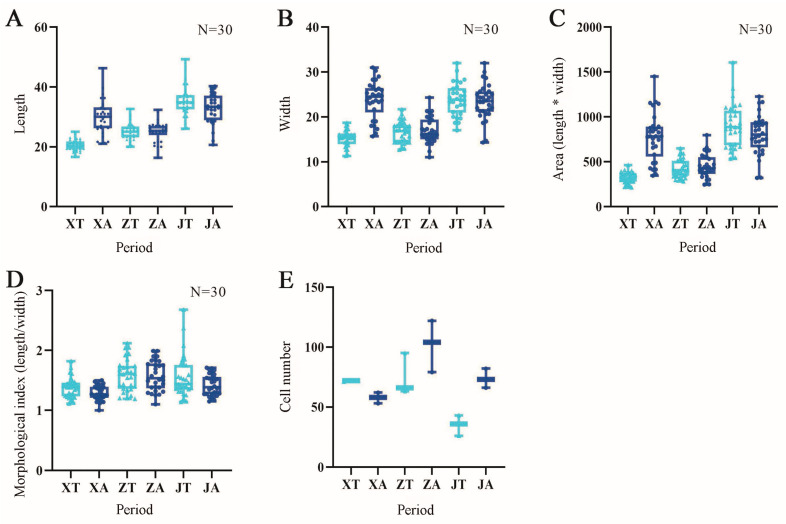
Determination of cell morphological indexes in three developmental stages of *A. carambola*. (**A**) Cell length changes in *A. carambola* fruit. (**B**) Cell width changes in *A. carambola* fruit. (**C**) Cell area changes in *A. carambola* fruit. (**D**) Cell morphology changes in *A. carambola* fruit. (**E**) Cell number changes in *A. carambola* fruit. XT, ZT, and JT represent the convex parts of the Y1, Y2, and Y3 periods of *A. carambola* fruit. XA, ZA, and JA represent the concave parts of the Y1, Y2, and Y3 periods of *A. carambola* fruit.

After the end of the flowering period, the fruit development stage was started. In the Y1 stage, the central diameter of the fruit was 3–4 mm, and the overall appearance showed that the small fruit at this time was surrounded by five pale pink sepals. The five edges of the fruit were angular, presenting a three-dimensional pentagram. Five styles and five filaments were loosely arranged and had red pedicels (Figure 2A). Compared with the flower bud stage, the ovary was fully developed into a pentagram shape, and compacted flesh cells started to appear. The arrangement of cells was more compact. At that time, the area of the central concave part of the fruit was larger than that of the cells at the sharp corner of the fruit. However, the cells at the sharp corner of the fruit were smaller and densely arranged, and the cell shape of both was square. In addition, the number of fruit vascular bundles increased significantly, and thick angular tissue appeared at the sharp corners to protect them (Figure 2A-1,A-2,A-3).

In the Y2 stage, the diameter of the middle part was 4–6 mm, which was not much different than the first stage of the fruit, except that the volume of the fruit increased significantly, the ovary occupied most of the fruit, and the peduncle gradually turned a pale green (Figure 2B). The slices showed that the size and number of cells in the convex part of the fruit did not change much, the cells in the depression area became smaller and more numerous, and the cells in the depression in the center of the fruit were not much different in size and shape from those in the sharp corners of the fruit. The cells tended to be elongated, but the overall shape was nearly square (Figure 2B-1,B-2,B-3).

In the Y3 stage, the diameter of the middle part of the fruit was 6–12 mm, and the ovary had become the main body of the fruit. The fruit volume was significantly larger than in the previous two periods (Figure 2C). The results of slicing showed that the cells were elongated, the number and size of the raised parts of the fruit decreased, and the cells in the depressed parts increased in number. The cells were also relatively sparsely arranged, with major vascular bundles beginning to appear in the direction of the carpel rays, distributed in multiple locations, and the seeds were becoming more mature (Figure 2C-1,C-2,C-3).

### 2.2. Analysis of Cell Morphology in Three Developmental Stages of the Fruit of A. carambola

The cells in the flower bud stage were too small to measure the stage-specific morphological index. By measuring the fruit morphological index of the *A. carambola* pentacle and analyzing the data visually, the phenotypic characteristics of the *A. carambola* pentacle were correlated with each other. The cell length showed an overall increasing trend in the convex and concave parts of the fruit and reached the maximum in the Y3 stage (Figure 3A). According to the variation amplitude of the *A. carambola* bulge and depression, the Y2 period was the key period. The cell length in the convex part of Y1 was obviously smaller than that in the concave part, while the growth law of the cell length in the Y2 period was the opposite. As a result, the cell length in the convex part of the Y2 period was the same as in the concave part. Additionally, there was minimal change in cell length in both of these regions. The changes in cell width were similar to that of cell length. From the beginning, the cell width of the convex part was larger than the cells in the concave part. However, in the end, there was no difference in the cell width of the two parts. The maximum cell width was observed during the Y3 period (Figure 3B). The cell area decreased during the Y2 period, while the difference between the convex and concave surfaces was maximum during the Y3 period (Figure 3C). The cell morphology index was the ratio of cell length to cell width. The elongation of cells was measured by the size ratio. The larger the cell morphology index, the more pronounced the cell elongation. The cell morphology index did not change significantly. The cell morphology index in the Y1 stage was close to one, with the length and width of the individual cells being similar. The cell morphology index increased in the Y2 and Y3 stages, indicating an appropriate cell elongation, until reaching the final morphology of the *A. carambola* fruit cells (Figure 3D). In terms of the number of cells, the convex part of the fruit exhibited a decreasing trend. However, the concave part showed a pattern of initially increasing and then decreasing the cell number. Overall, in the Y1 stage, the number of cells in the convex part of the fruit was greater than that in the concave part, while in the Y2 and Y3 stages, the number of cells in the convex part was smaller than that in the concave part (Figure 3E).

### 2.3. Ultramicroscopic Observation of the Development Process from Bud to Blossom of the A. carambola Fruit 

**Figure 4:**
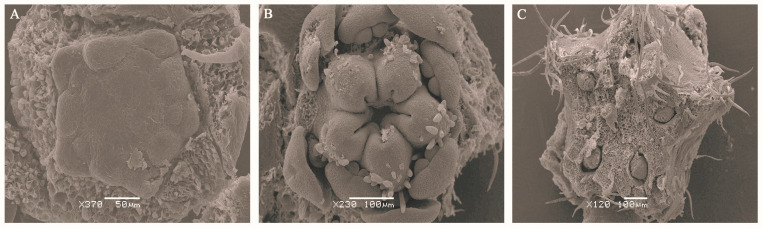
Scanning Electron Microscopy (SEM) observation of epidermal cells in three developmental stages of *A. carambola* ovary. (**A**) SEM observation of y1 stage of *A. carambola* bud. (**B**) SEM observation of y2 stage of *A. carambola* bud. (**C**) SEM observation of y3 stage of *A. carambola* bud.

To further clarify the microscopic process of pentagonal fruit formation, the ovary development of *A. carambola* at different developmental stages was observed by scanning electron microscopy. In the y1 stage, the sepals, petal primordia, stamen primordia, and pistil primordia were fully formed. The ovary was smooth, the cells were tightly arranged, and the ovary was nearly round. (Figure 4A). With the development of the flower bud, in the y2 stage, the sepals were clearly visible, and the petals, stamens, and pistils continued to develop. It could be clearly observed that the ovary at this time had the shape of a pentagram, and there were a few accessory hairs (Figure 4B). In the y3 stage, the ovary was fully developed in the shape of a three-dimensional pentagram. In this stage, five embryo sacs were also observed, and the hair growth was significantly increased in this stage (Figure 4C).

### 2.4. Endogenous Hormone Changes in Fruit Development of A. carambola

**Figure 5:**
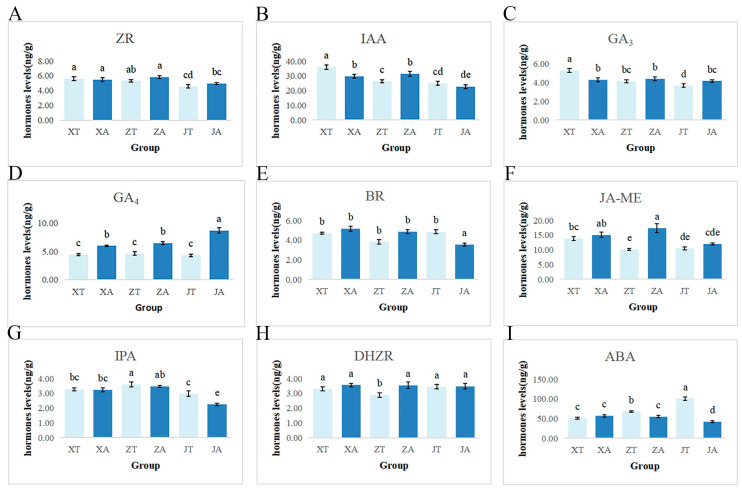
Changes in auxin content in *A. carambola* fruit at different periods. XT, ZT, and JT represent the convex parts of the Y1, Y2, and Y3 periods of *A. carambola* fruit. XA, ZA, and JA represent the concave parts of the Y1, Y2, and Y3 periods of *A. carambola* fruit. Results of the group comparisons are shown. (**A**) ZR content map. (**B**) IAA content chart. (**C**) GA3 content map. (**D**) GA4 content map; (**E**) BR content map. (**F**) JA-ME content map. (**G**) IPA content chart. (**H**) DHZR content map. (**I**) ABA content chart. Groups with different letters indicate statistically significant differences (*p* < 0.05). Statistical analysis was performed using one-way ANOVA followed by multiple comparison tests.

The contents of nine endogenous hormones in the *A. carambola* fruit were determined and it was found that the contents of IAA and ABA fluctuated greatly in general, while the contents of Zeatin (ZR), GH3, Gibberellin Acid 4 (GA4), Brassinosteroids (BR), Jasmonic Acid Methylester (JA-ME), Indolepropionic acid (IPA), and Dihydrozeatin riboside (DHZR) did not change significantly (Figure 5). This indicates that IAA and ABA play a more dynamic role in fruit development compared to the other hormones. In the first stage, the content of IAA in the convex part was significantly higher than that in the concave part. With the development of the fruit, there was a turning point in the second stage, and the hormone content in the concave part was higher than that in the convex part. In the third stage of the development of the fruit, the content of IAA in the convex part was higher than that in the concave part (Figure 5B). The ABA content in the convex part was lower compared to the depression part in the first stage, and the content in the convex part was significantly higher than that in the concave part in the second and third stages (Figure 5I). Although the hormone content of ZR, GA3, GA4, BR, JA-ME, and DHZR did not change significantly, the concave part showed a higher trend than the convex part in the three periods (Figure 5A,C,E,H). This suggests a potential localized accumulation of these hormones in the concave part. The hormone content of IPA showed an opposite trend, with the concave part having a lower IPA content than the convex part (Figure 5G).

### 2.5. Transcriptome Sequencing Screened the Regulatory Genes Related to the Growth and Development of A. carambola Fruit Shape

**Figure 6:**
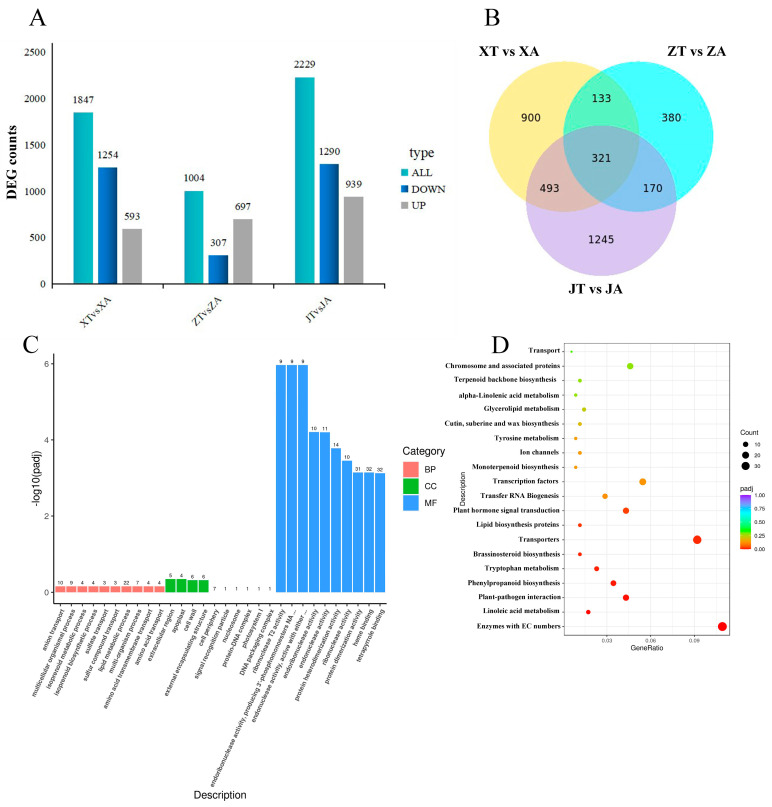
Transcriptomic analysis of *A. carambola* fruit. (**A**) Statistical analysis of the results of the DEGs (*q* < 0.05). (**B**) Differential gene Ven diagram. (**C**) Enriched Gene ontology (GO) terms of DEGs between ZT and ZA. (**D**) KEGG enrichment map of DEGS between ZT and ZA.

Based on the screening criterion of differentially expressed genes (padj ≤ 0.05), the number of differentially expressed genes was almost equal between the XT vs. XA combination and the JT vs. JA combination and was greater than that of the ZT vs. ZA combination (1847 and 2229, respectively). The number of differential genes between the JT and JA combinations was the highest, with 2229 Differentially Expressed Genes (DEGs), including 939 up-regulated and 1290 down-regulated genes. The number of differential genes between the ZT and ZA combinations was the least (Figure 6A). There was a total of 321 co-expressed genes among the three groups of samples (Figure 6B). A total of 1245 genes were expressed between the JT and JA combinations, 900 between the XT and XA combination, and the least (380) were expressed between the ZT and ZA combinations.

Based on the previous series analysis of differential gene expression in the pentagram fruit of *A. carambola*, the combination ZT vs. ZA in the transitional period was selected for a gene functional enrichment analysis. Differential genes were significantly enriched in molecular function (MF) and biological process (BP), with a relatively small amount of enrichment in the cell component (CC) category (Figure 6C). In molecular function, the items with significant enrichment were mainly the DNA packaging complex, ribonuclease T2 activity and endoribonuclease activity, protein heterodimerization activity, ribonuclease activity, protein dimerization activity, and heme binding. Anion transport and lipid metabolic processes were the main biological processes. The items rich in cell components included the external encapsulating structure, apoplast, and cell wall.

A Kyoto Encyclopedia of Genes and Genomes (KEGG) pathway enrichment analysis was performed on the combination ZT vs. ZA during the transition period. It was found that the differential genes were mainly enriched in the Enzymes with EC numbers, Transporters, Transcription factors, Plant hormone signal transduction, and other items (Figure 6D).

### 2.6. AcaAUX/IAA, AcaGH3, AcaYAB, AcaXTH and AcaEXP Gene Expression Patterns

**Figure 7:**
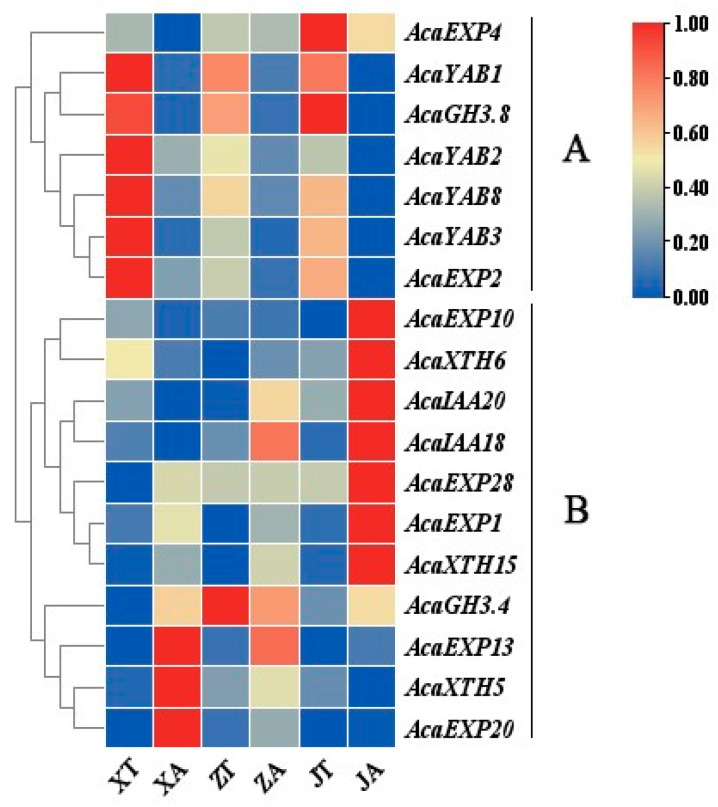
Expression analysis of related functional genes in the pentagram fruit of *A. carambola*. The expression levels are represented by the colors: red shows up-regulated, and blue shows down-regulated gene expression.

The fragments per kilobase per million (FPKM) values of *AcaAUX/IAA*, *AcaGH3*, *AcaYAB*, *AcaXTH*, and *AcaEXP* genes were further analyzed by reverse blast, smart, and CDD. The qualified sequences were heat-mapped by TBtools after the removal of the redundant, incomplete domain and low expression sequences. A total of 18 genes showed significant variations across the developmental stages. These genes were divided into two groups, A and B. Group A included *AcaEXP4*, *AcaYAB1*, *AcaGH3.8*, *AcaYAB2*, *AcaYAB8*, *AcaYAB3*, and *AcaEXP2* genes, and group B included *AcaEXP10*, *AcaXTH6*, *AcaIAA20*, *AcaIAA18*, *AcaEXP28*, *AcaEXP1*, *AcaXTH15*, *AcaGH3.4*, *AcaEXP13*, *AcaXTH5*, and *ACAEXP20* genes. The expression of A group genes was significant in the convex part of the *A. carambola* fruit, showing almost no expression in the concave part. Their expression gradually decreased with the increase in the development of fruit. Contrarily, the expression of group B genes increased gradually with the development of fruit. However, the expression levels of four genes, *AcaGH3.4*, *AcaEXP13*, *AcaXTH5*, and *AcaEXP20*, in the group B genes gradually decreased with the development of fruits (Figure 7).

### 2.7. Analysis of Expression Patterns of Related Genes

**Figure 8:**
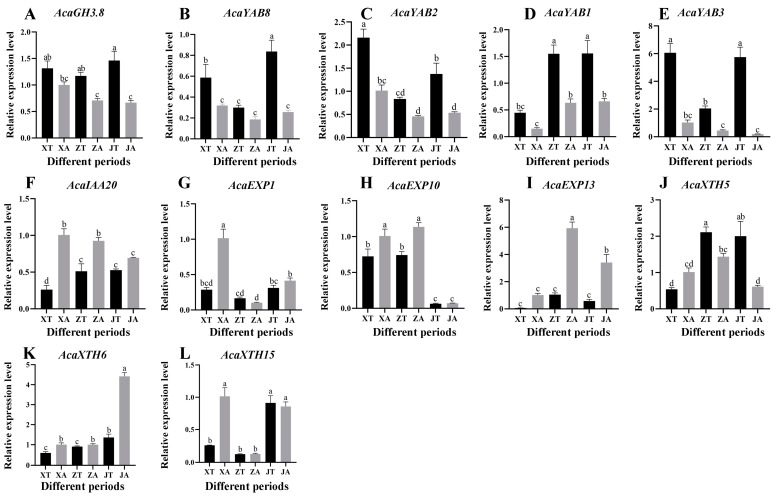
Analysis of the expression of key genes in different stages of *A. carambola* fruit development. (**A**) Expression of *AcaGH3.8* at different stages. (**B**) Expression of *AcaYAB8* at different stages. (**C**) Expression of *AcaYAB2* at different stages. (**D**) Expression of *AcaYAB1* at different stages. (**E**) Expression of *AcaYAB3* at different stages. (**F**) Expression of *AcaIAA20* at different stages. (**G**) Expression of *AcaEXP1* at different stages. (**H**) Expression of *AcaEXP10* at different stages. (**I**) Expression of *AcaEXP13* at different stages. (**J**) Expression of *AcaXTH5* at different stages. (**K**) Expression of *AcaXTH6* at different stages. (**L**) Expression of *AcaXTH15* at different stages.

The 12 DEGs with the most obvious differential expression and complete domains were selected, and the key genes with significant differential expression were further validated by RT-qPCR. The RT-qPCR expression of most genes was basically consistent with the general trend of the transcriptome expression pattern. The expression levels of *AcaGH3.8*, *AcaYAB8*, *AcaYAB2*, *AcaYAB1*, and *AcaYAB3* were higher in the convex part than in the concave part in the three stages of *A. carambola* fruit development (Figure 8A–E). The other seven genes, namely *AcaIAA20*, *AcaEXP1*, *AcaEXP10*, *AcaEXP13*, *AcaXTH5*, *AcaXTH6*, and *AcaXTH15*, all showed the opposite expression trend (Figure 8F–L). In short, during the three developmental stages of the *A. carambola* fruit, the expression level in the concave part was higher than that in the convex part of the *A. carambola* fruit.

**Figure 9:**
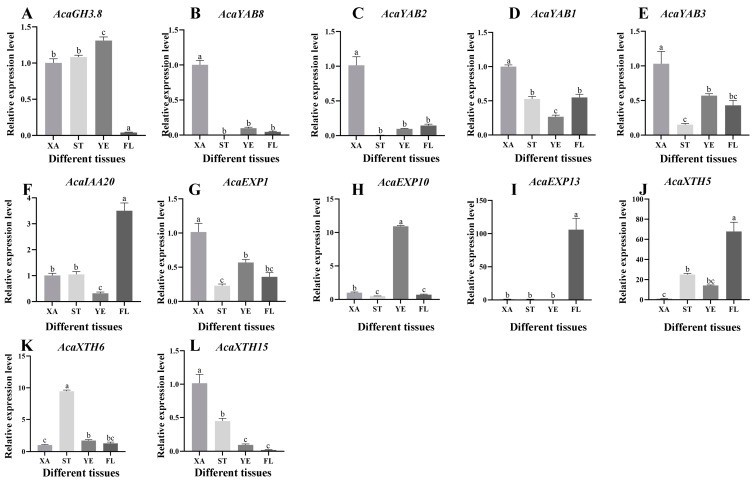
Analysis of the expression of key genes in different tissues of *A. carambola* fruit. (**A**) Expression of *AcaGH3.8* at different tissues. (**B**) Expression of *AcaYAB8* at different tissues. (**C**) Expression of *AcaYAB2* at different tissues. (**D**) Expression of *AcaYAB1* at different tissues. (**E**) Expression of *AcaYAB3* at different tissues. (**F**) Expression of *AcaIAA20* at different tissues. (**G**) Expression of *AcaEXP1* at different tissues. (**H**) Expression of *AcaEXP10* at different tissues. (**I**) Expression of *AcaEXP13* at different tissues. (**J**) Expression of *AcaXTH5* at different tissues. (**K**) Expression of *AcaXTH6* at different tissues. (**L**) Expression of *AcaXTH15* at different tissues.

In addition to analyzing the expression levels of the protrusion and depression of *A. carambola* during different periods, the expression of these 12 genes in the stems, leaves, and flowers of *A. carambola* was also verified during the first period (XA). When compared with the depression of the *A. carambola* pentagonal fruit, half of the genes showed lower expression in the stem, leaf, and flower of the *A. carambola* fruit. The expression of the *AcaIAA20*, *AcaEXP13*, and *AcaXTH5* genes was very high in the flowers (Figure 9F–H), and the expression of *AcaEXP13* was significantly low in the concave parts of the *A. carambola* fruit, stems, and leaves (Figure 9I). The *AcaGH3.8*, *AcaEXP10*, and *AcaXTH6* genes were expressed in the four tissues to varying degrees (Figure 9A,H,K), but the expression levels of *AcaGH3.8* and *AcaEXP10* were the highest in leaves (Figure 9A,H), while the expression levels of *AcaXTH6* were the highest in stems (Figure 9K).

### 2.8. Key Gene Cloning and Subcellular Localization

The CDS sequences of six genes, *AcaGH3.8*, *AcaIAA20*, *AcaYAB2*, *AcaXTH6*, *AcaYAB3*, and *AcaEXP13*, were screened according to the genome of *A. carambola*, and PCR amplification was performed using gene-specific primers. Products with lengths of 1812 bp, 1098 bp, 651 bp, 906 bp, 537 bp, and 768 bp were obtained, and the PCR pattern is shown in Figure S4.

**Figure 10:**
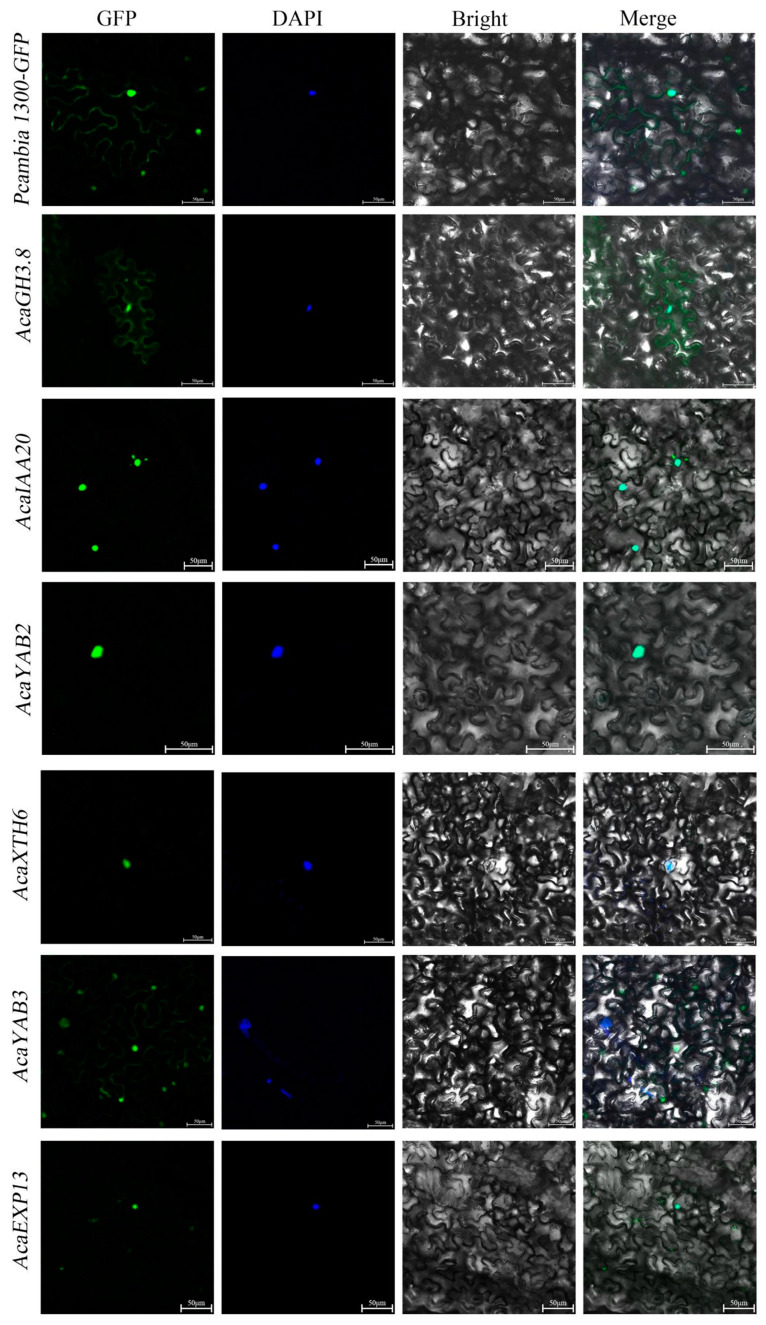
Subcellular localization of *AcaGH3.8*, *AcaIAA20*, *AcaYAB2*, *AcaXTH6*, *AcaYAB3*, and *AcaEXP13* in *A. carambola*.

The obtained homologous recombinant products were transformed into *Escherichia coli* receptive cells. *AcaGH3.8* was primarily located in the cytoplasm and nucleus. *AcaIAA20*, *AcaYAB2*, and *AcaXTH6* were primarily located in the nucleus. *AcaYAB3* was mainly located in the nucleus and cell membrane. *AcaEXP13* was positioned in the plasma membrane (Figure 10).

## 3. Discussion

The microscopic examination of the cell morphology of fruit cells in the three developmental stages of the *A. carambola* fruit suggested cell number plays a major role in the morphological development of *A. carambola* pentagonal fruit. Our finding was consistent with the fruit development pattern of *C. sativus*, *C. melo* [10], *P. avium* [11], *P. persica* [12], and *Pyrus* spp. [12]. Cell division in the early stage increases the number of cells, and it is also the fruit cells in the early stage that develop into the final fruit shape and form the prototype of a pentagram. Moreover, the accumulation of cells in this stage lays the foundation for the expansion of cells in the next stage [51]. It is the difference in the number of cells that leads to the difference in the shape of the convex and concave parts of the pentagram. This is consistent with the results indicating that the arrangement of paraxial and distal cells in *A. thaliana* leaves is different due to the establishment of paraxial and distal polarity [33], which reflects the characteristics of cell polarity in morphology. At the same time, there are protrusions in the carpel primordium during the flower bud Y1 stage, which contain stigmas and flower columns, indicating that the fruit shape of *A. carambola* was built during the development of the ovary before flowering.

The polarity phenomenon caused by the difference in cell number in the *A. carambola* fruit phenotype may be related to the gradient distribution of hormones caused by the polar transport of endogenous auxin [52]. The contents of nine kinds of endogenous hormones in the convex and concave parts of *A. carambola* were analyzed and measured from the physiological aspect. Among them, the IAA content in the convex and concave parts of the *A. carambola* fruit showed a certain regularity. The IAA content was higher during the protrusion than during the depression in the first and third periods. The formation of this gradient is the result of the combined action of auxin synthesis in vivo and polarity transport between cells [52,53]. This is manifested as differences in the number of cells, which is different from the role of auxin polar transport in tobacco leaves in regulating cell elongation [54]. Similar polarity phenomena were studied in leaves [55], petals [56], and sepals, but further studies are needed to confirm the polarity phenomena in the fruit of *A. carambola*.

The auxin release signal is affected by related signal pathways and functional genes and indirectly regulates the *A. carambola* fruit shape by influencing the gene expression level. A transcriptome sequencing analysis suggested the functional genes related to fruit shape, and AUX/IAA and GH3 gene families were directly related to auxin distribution. *AcaIAA18* and *AcaIAA20* belong to the AUX/IAA family, which negatively regulate growth proteins, and the *SlIAA9* gene encodes transcription suppressors to regulate the occurrence of tomato leaf shape and fruit setting [26]. In addition, an overexpression of *IAA20* in *A. thaliana* also leads to frequent compromises in the direction of gravity growth of hypocotyls and roots [57]. As the homologous genes of *AtIAA9* and *SlIAA9*, *AcaIAA18* and *AcaIAA20* have higher expression levels in the concave part of *A. carambola*, but lower expression levels in the convex part. Contrary to the changes in hormone content and cell number, they reflect the negative regulation of *AcaIAA18* and *AcaIAA20* and promote the establishment of a polarity axis in the convex and concave parts.

GH3 and AUX/IAA are collectively referred to as auxin early response genes, which may affect the distribution of hormones in response to auxin and thus regulate plant morphology. In this study, the expression levels of *AcaGH3.4* and *AcaGH3.8* in the convex site were significantly higher than in the concave site. The expression pattern of the GH3 gene was exactly opposite to that of AUX/IAA, which may be a feedback regulation effect of the GH3 catalysis on the amination of IAA, thereby maintaining the auxin balance [58]. The transcript of a *GH3* gene (*GH3-1*), encoding for an IAA-amido synthetase that conjugates IAA to amino acids, was detected in grape berries (*Vitis vinifera*), and it can play a role in early fruit ripening and development [59]. It is speculated that *AcaGH3.4* and *AcaGH3.8* may be involved in regulating the elongation of fruit cells, and this effect is stronger in the convex part.

In addition to auxin, YABBY is considered to be a key gene regulating cell polarity and its function is to promote leaf expansion in response to near-distal polarity [39,60]. The expression levels of *AcaYAB2* and *AcaYAB3* in the convex part of *A. carambola* were significantly higher than in the concave part. This suggests that the *AcaYAB* gene plays a certain role in *A. carambola* fruit polarity, which is similar to the function of the YABBY gene in the *Citrus medica* ‘Fingered’ [50]. How it affects the polarity of meristem needs further exploration.

Plant cell wall relaxation and cell elongation are the basis of fruit cell growth, and the synergistic effect of the *AcaXTH* and *AcaEXP* genes can control cell wall relaxation and cell elongation [61]. In *A. thaliana*, *AtXTH9* can elongate flower buds and stems [62], and *AtEXP10* is related to the enlargement of leaf and petiole cells [63]. The expression levels of the *AcaXTH* and *AcaEXP* genes are higher in the concave part of the *A. carambola* fruit than in the convex part. It is inferred that the elongation of the concave part is stronger than that of the convex part and this elongation and enlargement effect is approximately consistent with the previous morphological indexes. That is, the different expression levels of genes in the convex and concave parts of *A. carambola* fruit may be related to the different effects and degrees of genes.

These findings suggest that *AcaIAA18* and *AcaIAA20* may play a crucial role in the feedback regulation of auxin distribution by modulating auxin biosynthesis, transport, or the signal transduction pathways. Specifically, these genes could influence auxin concentration gradients by altering the expression of other genes involved in auxin polar transport, such as PIN-FORMED (PIN) proteins, or by modulating the activity of auxin response factors (ARFs), which are central to auxin signaling [64]. This complex regulatory network likely contributes to the differential auxin levels observed between the convex and concave parts of the *A. carambola* fruit. For example, in *Arabidopsis*, the AUX/IAA and GH3 gene families are known to regulate the auxin levels and distribution, affecting organ polarity and growth patterns [64]. A similar mechanism could be operating in *A. carambola*, where the differential expression of *AcaIAA18* and *AcaIAA20* may establish the auxin gradients that drive the distinct morphological features of the fruit. GH3 genes, which respond to auxin, may balance hormone levels by conjugating excess auxin, preventing overaccumulation. *AcaGH3.4* and *AcaGH3.8*, for example, have higher expression in the convex regions, potentially promoting cell elongation. Additionally, YABBY genes, which regulate cell polarity, show higher expression in the convex regions, suggesting their role in fruit shape development. Meanwhile, *AcaXTH* and *AcaEXP* genes, which control cell wall relaxation and elongation, are more expressed in the concave regions, aligning with the observed morphological differences.

Moreover, the differential auxin distribution, mediated by these gene families, likely triggers localized changes in cell proliferation and expansion, contributing to the differences in cell number between the convex and concave regions. This aligns with the hypothesis that auxin gradients, established through the coordinated regulation of gene expression, are key determinants of fruit shape in *A. carambola*. Such a mechanism is reminiscent of how auxin regulates tissue development in other plant organs, such as leaves and flowers [65]. Therefore, understanding the interplay between gene expression and hormone levels provides crucial insights into the molecular basis of fruit morphogenesis in *A. carambola*.

**Figure 11:**
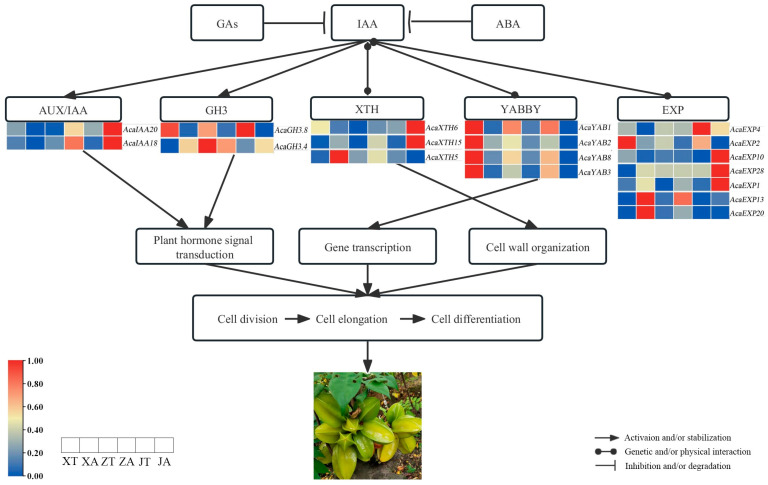
Regulatory expression network of the pentagram of *A. carambola* fruit.

In summary, this study explores the potential mechanisms underlying the pentagram shape of *A. carambola* fruit by examining the relationship between fruit morphology, hormone content, and transcriptome data (Figure 11). The hypothesis proposed suggests that the pentagram shape is influenced by the polar development genes (AUX/IAA, GH3, YABBY) and cell development genes (XTH, EXP), which exhibit differential expression. These genes are thought to regulate auxin signaling and thereby contribute to hormone concentration gradients. Consequently, the distribution of fruit cells appears to follow a regular pattern, resulting in the observed pentagram shape with both concave and convex surfaces.

This study provides a preliminary analysis of the genes responsible for the differences between the protruding and depressed parts of the *A. carambola* fruit but does not address whether the fruit originates from a rounded or pentacle-shaped ovary. Future research should use laser capture microdissection to better understand the fruit formation mechanisms. Additionally, since only nine endogenous hormones were measured, incorporating metabolomics to explore a broader range of hormones and their regulatory roles is recommended. Further, although transcriptomics and quantitative PCR identified key regulatory genes, functional validation is needed. Techniques such as gene silencing or exogenous hormone treatments could be used for this purpose. Extending these methods to other plants could provide insights into the universal fruit development mechanisms and support crop improvement.

## 4. Materials and Methods

### 4.1. Plant Materials

The flower buds and fruits of a three-year-old ‘Daguo Tianyangtao 1’ grown in the A. carambola Germplasm Resource Nursery (108.25′15′′ E, 22.85′83′′ N) of the Institute of Horticulture at the Guangxi Academy of Agricultural Sciences were used to collect samples in September 2021 (Figure S1). Plants with good growth conditions and consistent phenotypes were selected for sampling. The sampling was performed between 10:00 a.m. and 11:00 a.m. every day. During the sampling period for the *A. carambola* flower bud, the transverse diameter of the ovary of the flower bud was taken as the standard. The flower buds were stripped under a stereomicroscope (Jiangnan, NSZ818). The peripheral floral organs were removed, leaving the ovary behind. The sampling period of the flower buds was divided into 3 periods: y1 (light green bud with a diameter < 0.1 cm), y2 (white–pink bud with a diameter < 0.1 cm), and y3 (red bud with a diameter 0.1 cm–0.2 cm). The fruit sampling was also performed in three stages. The Y1 stage included fruits with a transverse diameter of 3–4 mm. The Y2 stage included fruits with a 4–6 mm diameter and the third stage (Y3) contained fruits with a transverse diameter of 6–12 mm.

### 4.2. Paraffin Section Sample Preparation and Observation

Referring to the experimental method of Fischer et al. [66], the *A. carambola* flower buds of the three periods after stereoscopic dissection were fully fixed in the fixative solution and fixed at 4 °C for 24 h. Before an analysis, flower buds were tertiary dehydrated with 70%, 85%, and 95% ethanol, respectively. Then at a temperature of 60–63 °C, the samples were fixed in paraffin wax and cut to 6 μm using a microtome. Tissue sections were stained with a crocus-solid green staining and observed under a light microscope (Nikon China, ECLEPSE Ci-L) (Figure S2).

### 4.3. SEM Sample Preparation and Observation

After dissecting the fresh *A. carambola* flower buds, they were immediately fixed with a Gluta fixative solution for 72 h. Then, the samples were rinsed with a phosphoric acid buffer 3 times, each time was 10–15 min apart. Samples were fixed with a 1% osmium acid solution for 4 h, and washed with distilled water 3 times at intervals of 10–15 min each time. Fractional dehydration (50%, 70%, 80%, 90%, 100%) was performed with ethanol of different gradients, each time separated by 10–15 min, and then replaced with 100% ethanol twice. After replacing it with a propylene oxide solution 2 times, Hitachi HCP-2 was dried at the critical point and sprayed with gold (EIKO IB-5). Finally, buds were observed under a scanning electron microscope (SEM, JEOL JSM-6380lv).

### 4.4. Image Analysis and Data Measurement

Three of the most representative slices were selected from the five materials (*A. carambola* flower buds SEM photographs) in each period, from which 30 cells with uniform size and complete and clear outlines in the same part and vision were randomly selected for morphological index determination. The IMAGE J 2.0.0 software measurement tool was used to count the fruits of *A. carambola* in the three different development periods. The cell length, width, and rough area were measured. In the later stage, Excel 2309, Statistical Product and Service Solutions (SPSS) Statistics 21.0, Photoshop CS6, and Graphpad Prism 9 were used for data processing and mapping. Among them, the *A. carambola* fruit was differentiated into convex and concave periods for data statistics, including the Y1 period (3– 4 mm): XT, XA, Y2 period (4–6 mm): ZT, ZA, and Y3 period (6–12 mm): JT, JA.

### 4.5. Hormone Content Determination

The experimental material used was the same as that used in the hormone sampling. The difference was that the convex and concave parts of *A. carambola* were distinguished and sampled, respectively, in this experiment, as shown in Figure S3. In the three different developmental stages, the convex parts were labeled as XT, ZT, and JT, corresponding to the 1st, 2nd, and 3rd stages. The concave parts were labeled as XA, ZA, and JA, corresponding to the 1st, 2nd, and 3rd stages. The concave and convex fruit parts were cut and stored in liquid nitrogen and stored at −80 °C for later use.

The enzyme-linked immunosorbent assay (ELISA) of plant hormones was used to determine the contents of nine endogenous hormones in the three developmental stages of the *A. carambola* fruit (Table S1). The specific methods of extraction, determination, and calculation of endogenous hormones are referred to in the operations of Yang et al. [67]. The specific steps are as follows: First, the sample was taken out of the −80 °C freezer, and 0.3 g was weighed and added to 2 mL of an extraction solution. The sample was ground in an ice bath to form a homogenate and then transferred to a test tube. Another 2 mL of extraction solution was used to rinse the sample, which was then transferred to the test tube, mixed thoroughly, and stored at 4 °C for 4 h. Next, the mixture was centrifuged at 3500 rpm for 8 min to collect the supernatant. Then, 1 mL of extract was added, and mixed well, and the extraction was continued at 4 °C for 1 h before centrifuging again. The supernatants were combined, and the residual material was removed. The supernatant was then filtered using a C-18 solid-phase extraction column, which was balanced with 80% methanol. This was followed by sample loading and washing with 100% methanol and ether, sequentially. Finally, the processed sample was transferred to a centrifuge tube, vacuum concentrated, or dried with nitrogen to remove methanol, and diluted to a constant volume with the sample dilution solution.

For sample determination, the standard samples were first prepared according to the dilution factors indicated on the labels, and a 2-fold serial dilution was performed based on the maximum concentration in the standard curve. Fifty microliters of each of the 8 concentration levels were added to a 96-well plate, including the test samples, with 2 replicates per concentration. Next, antibodies were added to the samples, 50 μL per well, and incubated at 37 °C for 30 min. The reaction solution was then discarded, and the wells were washed 4 times with washing solution. After that, enzyme-labeled secondary antibodies were added, and the plate was incubated at 37 °C for 30 min, followed by a repeat of the washing step. During color development, o-phenylenediamine (OPD) and hydrogen peroxide were dissolved into the substrate buffer, and the mixture was added to the samples. The reaction was stopped by adding sulfuric acid after the appropriate color development. Finally, the OD values at 490 nm were measured using an enzyme-linked immunosorbent spectrophotometer (Thermo Fisher Scientific, NanoDrop 2000/2000c), and the logit of the color development values was calculated using the ELISA method; the results were calculated using the logit curve, the abscissa of the curve was expressed as the natural logarithm of each concentration of the hormone standard (ng/mL), and the ordinate was expressed as the logit value of the chromogenic value of each concentration. The logit value is calculated as follows:

$$Logit(\frac{B}{B0})=ln\frac{\frac{B}{B0}}{1-\frac{B}{B0}}=ln\frac{B}{B0-B}$$

B0 represents the chromogenic value for the wells with a concentration of 0 ng/mL, while B denotes the chromogenic values for the other concentrations. The natural logarithm of the hormone concentration (ng/mL) in the sample is determined from the graph using the logit value of the sample’s color development. The hormone concentration (ng/mL) is then obtained by taking the antilog of this value. Finally, the hormone content in the sample (ng/g·fw) is calculated.

Data processing was performed with Excel, and one-way ANOVA was conducted using SPSS 26.0. Prism and Photoshop were used for plotting and image processing. For ANOVA, LSD was used under the equal variance assumption, and Tamhane T2 when the variances were unequal, with a significance level of 0.05.

### 4.6. RNA Extraction and Transcriptomic Sequencing (RNA-seq)

The total RNA from different parts of the *A. carambola* fruit was extracted using an RNA prep Pure plant Kit (TIANGEN, Beijing, China).

After the quality check, the total RNA was sent to Novogene (Beijing, China) for library construction and sequencing. In the sequencing experiment, two biological replicates were set for each group of samples. The library was first constructed, then diluted to 1.5 ng/μL, and the effective concentration of the library was measured by RT-qPCR. After the quality inspection, the samples were sequenced using Illumina NovaSeq 6000 (Illumina, San Diego, CA, USA). The transcriptome data were annotated and analyzed using the *A. carambola* genome as a reference [4]. After obtaining clean reads, the index of the reference genome was constructed using HISAT2 v2.0.5, and the paired end clean reads were compared with the *A. carambola* reference genome sequence. The number of reads covered by each gene from inception to termination was calculated using Feature counts. Then, the FPKM value of each gene was calculated based on gene length. A principal components analysis (PCA) was performed on all samples. Using the DESeq2 software (1.20.0), the differential expression between the two comparison combinations was analyzed to obtain a value of FDR (False discovery rate) (error discovery rate, padj being its common form). |log2 (Fold change)|> 1 and padj ≤ 0.05 were used as screening criteria for different expression genes (DEGs) [68].

Cluster Profile software 4.13.3 was used to perform a GO functional enrichment analysis and a KEGG pathway enrichment analysis for the differential gene sets and padj < 0.05 was used as the threshold for significant enrichment.

### 4.7. qRT-PCR

Using RNA as the template, the first strand of cDNA was synthesized by a reverse transcription kit (Takara Company, Tokyo, Japan, PrimeScriptTM IV 1st strand cDNA Synthesis Mix) and stored in the refrigerator at −20 °C for future use.

CDS-specific primers of *AcaIAA*, *AcaGH3*, *AcaYAB*, *AcaXTH*, and *AcaEXP* were designed using the Premier 5 for 12 genes. The *A. carambola* α-TUB [69] gene was used as the reference to calibrate the cDNA template quantities of different samples. The primer parameters were set as follows: Tm value of 58–61 °C, 23–25 bp primer length, and 45–60% GC content. Then it was sent to the Shanghai Bioengineering Co., Ltd. (Shanghai, China) for PAGE purification and synthesis of the primers (Table S2). The steps of real-time fluorescence quantification were carried out according to the operation instructions of the Taq Pro Universal SYBR qPCR Master Mix kit (Vazyme Biotech, Nanjing Co., Ltd., Nanjing, China), in which the internal reference was α-TUB.

### 4.8. Cloning and Subcellular Localization of Key Genes

The RNA of XT, ZA, JT, and JA of the *A. carambola* fruit were used as experimental materials for reverse transcription and the preparation of the first cDNA strand using the Hifair^®^ III 1st Strand cDNA Synthesis Kit (Yeason, Shanghai Co., Ltd., Shanghai, China). The tobacco seeds were stored in our laboratory.

According to the restriction sites of the *pCAMBIA 1300-GFP* vector, specific primers containing BamHI and StuI restriction sites were designed at both ends of the open reading frame of the gene (Table S3). Using the cDNA as the template, the 2× Hieff Canace^®^ Plus PCR Master Mix kit (Yeason, Shanghai Co., Ltd., Shanghai, China) was used for PCR amplification. The PCR amplification product was detected by Gel electrophoresis with 1% agarose, and then the Gel Extraction Kit was cut by the MolPure^®^ Gel Extraction Kit (Yeason, Shanghai Co., Ltd., Shanghai, China). The carrier linearization of circular *pCAMBIA 1300-GFP* was carried out using StuI and BamHI enzymes. The carrier was digested at 37 °C for 15 min. The plasmid that had not been digested was used as a marker for gel electrophoresis detection.

The linearized carrier was recovered using the glue-cutting recovery kit. The purified plasmid was then subjected to a homologous recombination reaction with the target gene fragment containing the cleavage site. The recombinant product was transformed into *Escherichia coli* DH5α receptor cells. After the bacteria grew on the plate, the monoclonal colonies were picked into an LB liquid medium containing 50 mg/L kanamycin and 600 μL LB with a sterilized gun tip and placed in a shaking table at 37 °C and 200 rpm for 4–6 h. The 1 μL bacterial solution was taken as the template for PCR amplification to detect whether there were target bands. The bacterial solution containing target bands was sent to the Shanghai Bioengineering Co., Ltd., in Shanghai, China, and double-ended sequencing was performed. After that, a sequence splicing, and comparison analysis were performed using DNAMAN 10 software. The bacterial liquid with the correct sequencing results was heavily shaken and then plasmid extraction was performed with the MolPure^®^ Plasmid Mini Kit (Yeason Biotechnology Shanghai Co., Ltd., Shanghai, China). Finally, the transformation and identification of *Agrobacterium* GV3101 was carried out.

The seeds of *Nicotiana benthamiana* were sown in the acupuncture tray in advance, and grew for 3–4 weeks. We configured the required concentration of the resuspension solution, activated the agrobacterium, and centrifuged the activated bacterial solution at 4000 rpm at room temperature (25 °C) for 10 min. Then, the solution was re-suspended and mixed with the pre-diluted infection solution (10 mM MES-KOH, pH = 5.6, 10 mM MgCl_2_, 100 uM AS) to measure the OD600 value at 0.6–0.8, and kept in dark at room temperature (25 °C) for 2 h. The bacterial solution was injected into tobacco and incubated under dark conditions for 48–72 h, and finally observed fluorescence imaging occurred under a laser confocal microscopy (Zeiss, Oberkochen, Germany, LSM880).

## 5. Conclusions

In conclusion, this study advances our understanding of the growth and development of *Averrhoa carambola*, particularly the formation of its distinctive pentagram-shaped fruit. The research demonstrates that cell number and size significantly contribute to fruit development, with the accumulation of different numbers of cells playing a dominant role. The fruit’s shape is determined before flowering, and its development involves maintaining the pentagram structure. A physiological analysis revealed that the polar transport of auxin, particularly IAA and ABA, plays a critical role in regulating the fruit’s shape, with higher concentrations observed in the raised parts of the fruit. This hormonal regulation is closely synchronized with the changes in cell morphology and phenotype. Furthermore, key genes potentially involved in this regulatory process, such as *AcaGH3.8*, *AcaIAA20*, *AcaYAB2*, *AcaXTH6*, *AcaYAB3*, and *AcaEXP13*, were identified through a transcriptome analysis, providing new insights into the genetic control of *A. carambola* fruit shape development.

## Data Availability

All relevant data can be found within the manuscript and its Supplementary Material.

## Supplementary Materials

The following supporting information can be downloaded at: https://www.mdpi.com/article/10.3390/plants13192696/s1, Figure S1: six sampling periods from bud to fruit development of *A. carambola*; Figure S2: *A. carambola* fruit shows concave and convex photograph; Figure S3: *A. carambola* fruit hormone sampling plot; Figure S4: PCR amplification of genes. Table S1: Abbreviates in this study; Table S2: sequence of gene-specific primers; Table S3: sequence of gene specific primers.
