# Supplementary material for: Developmental Morphology, Physiology, and Molecular Basis of the Pentagram Fruit of Averrhoa carambola
Source: Plants (Basel). 2024 Sep 26;13(19):2696. doi: 10.3390/plants13192696 (PMC11478451; doi:10.3390/plants13192696)
Supplement: Supplementary file 1 [file plants-13-02696-s001.zip › plants-3192967-supplementary.pdf]

Supplementary tables:

Table S1. Abbreviates in this study.

Abbreviates	English name
ZR	Zeatin
IAA	Auxin
GAs	Gibberellin Acid
BR	Brassinosteroids
JA-ME	Jasmonic Acid Methylester
IPA	Indolepropionic acid
DHZR	Dihydrozeatin riboside
ABA	Abscisic acid

Table S2. Sequence of gene specific primers.

Gene name	Forward primer	Reverse primer
α-TUB	CTTGGTGTTTAGTGCCGTTG	CCTGAGGAGAAGGATAGATGGT
AcaIAA20	GCCACCAGCGGAACTAACCTTATG	GCCAACCAACAACTTGTGCCTTG
AcaGH3.8	AGCAACAACAACAGCAATGGAATCG	AAGCGTCTCCGTTTGAACTTCTCC
AcaYAB8	CTGTCTTAGCGGTGAGTGTTCCTTG	CCACGCATATTCACAGGCAGGAG
AcaYAB2	CAATGATGCCGATTCGAGGAGGAG	GCGGTTGTAAGCAGATGGGACTC
AcaYAB1	AATCTGTGGTCCGTGAACATGGC	TGGGTGCTCGCATTGACATCTTG
AcaYAB3	TCGCTGCTTCCTTTCACTCAATGTC	CGGTTGTAGTAGGTGCTCGGTTTG
AcaXTH5	GGAGTGCCTATCAAGCCCTTAATGC	GGTGGAGGTACTGGGTATCGAGATC
AcaXTH6	AGACCCTTACCCTGCCTGTGTATC	ACCTCACAGCATCCTGGCAATAATC
AcaXTH15	CCTATCTCCGCCTCTCCGTTCC	GAATGGTCTCTCTTGTGGTCCTTGC
AcaEXP1	CAACTGGATGCAAATGAGCCGTAAC	GTTCATAGAAGTGGAGGTGCGTCTG
AcaEXP10	CGTAGGAGGTGCTGGTGATGTTC	ACTGGCGGTGATTTGAAATGAGAGG
AcaEXP13	CACTCAATCCCGAGCAGCTTACTAC	ACAGCCAAGGCCATTGCGATAG

Table S3. Sequence of gene specific primers.

Gene name	Forward primer
AcaGH3.8-F	ctccccttgctccgtggatccATGATGAGTAGCAACAACAACAGCA
AcaGH3.8-R	ctcgcccttgctcacaggcctGCTATATGCAGTGCTGTGAAACCT
AcaIAA20-F	ctccccttgctccgtggatccATGTCTCCGCCATCACCGT
AcaIAA20-R	ctcgcccttgctcacaggcctGTTTCTGTTCTTGGATTTCTCCATT
AcaYAB2-F	ctccccttgctccgtggatccATGTCCTCCTCATCATCTGCTTTT
AcaYAB2-R	ctcgcccttgctcacaggcctGTAAGGAGTGACACCCACATTTGTA
AcaXTH6-F	tctccccttgctccgtggatccATGGAGAGACGGGTCTCTTCAATGGCAAC
AcaXTH6-R	tcctcgcccttgctcacaggcctATCCCATGGACTCAATGTGCACTCAACAGG
AcaYAB3-F	ctccccttgctccgtggatccATGGCAAACCCTAATGGTGCT
AcaYAB3-R	ctcgcccttgctcacaggcctCTTGACAGCGATTGCACTTACATC
AcaEXP13-F	ctccccttgctccgtggatccATGGCTCTTTCTCTCAAGTCCCT
AcaEXP13-R	ctcgcccttgctcacaggcctTAAGTTGACTTGAACTCCTGTGTCATAA

Supplementary figures:

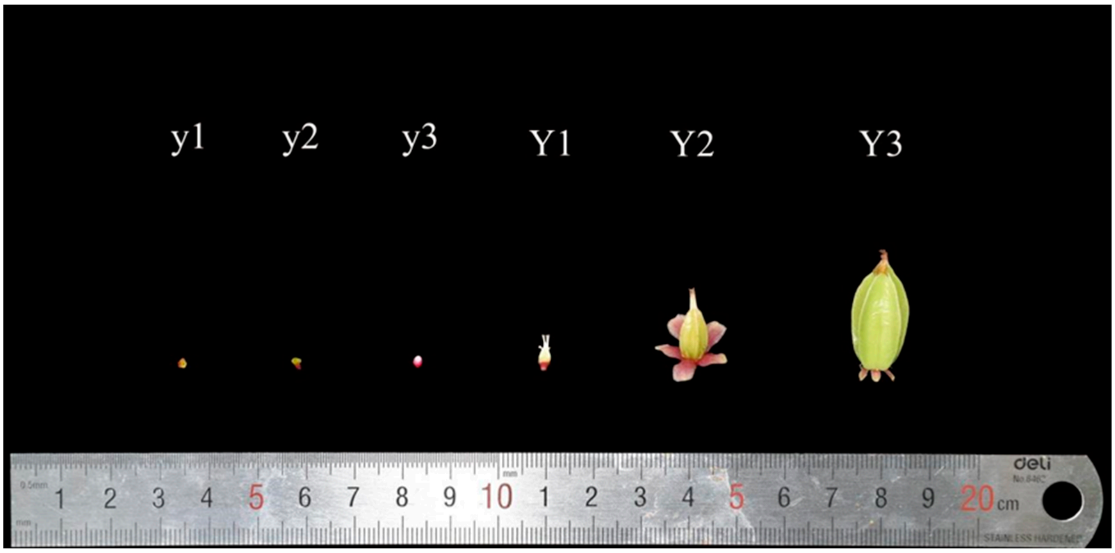

Figure S1. Six sampling periods from bud to fruit development of *A. carambola.* y1-y3. The sampling period of *A. carambola* blossom; Y1-Y3. The sampling period of *A. carambola* fruit

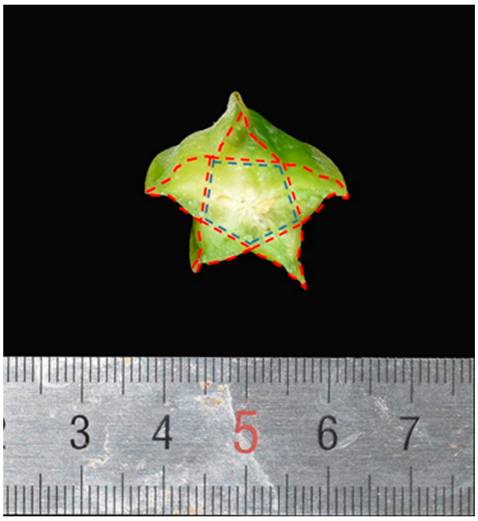

Figure S2. *A. carambola* fruit shows concave and convex photograph. The range circled by the red line is the convex, and the range circled by the blue line is the concave

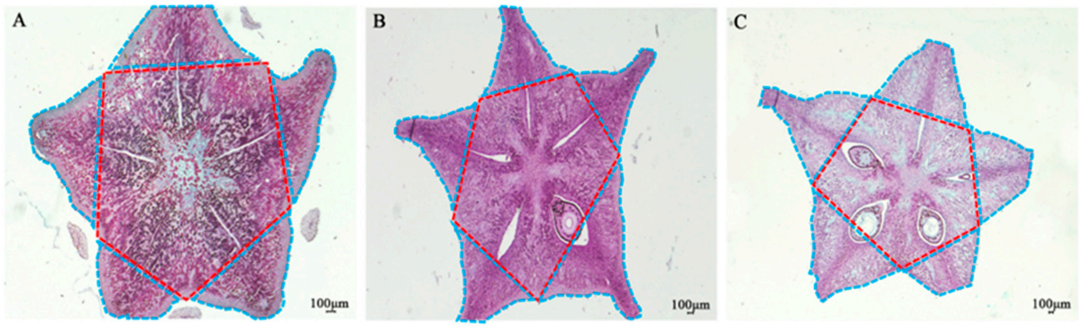

Figure S3. *A. carambola* fruit hormone sampling plot. The range circled by the red line is the concave, and the range circled by the blue line is the convex; A. Sample of *A. carambola* fruit at stage 1; B. Sample of *A. carambola* fruit at stage 2; C. Sample of *A. carambola* fruit at stage 3

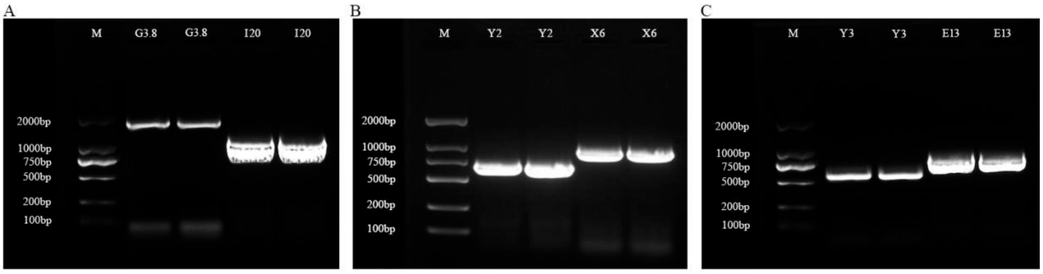

Figure S4 PCR amplification of genes. M is 2,000 bp Marker; G3.8, I20 are *AcaGH3.8* and *AcaIAA20* PCR product bands; Y2, X6 are *AcaYAB2* and *AcaXTH6* PCR product bands; Y3, E13 are *AcaYAB3* and *AcaEXP13* PCR product bands.
